# *ruvA* and *ruvB* mutants specifically impaired for replication fork reversal

**DOI:** 10.1111/j.1365-2958.2008.06431.x

**Published:** 2008-09-15

**Authors:** Marie Le Masson, Zeynep Baharoglu, Bénédicte Michel

**Affiliations:** 1 CNRS, Centre de Génétique MoléculaireUPR 2167, Gif-sur-Yvette, F-91198, France; 2 Université Paris-SudOrsay, F-91405, France; 3 Université Pierre et Marie Curie-Paris 6Paris, F-75005, France

## Abstract

Replication fork reversal (RFR) is a reaction that takes place in *Escherichia coli* at replication forks arrested by the inactivation of a replication protein. Fork reversal involves the annealing of the leading and lagging strand ends; it results in the formation of a Holliday junction adjacent to DNA double-strand end, both of which are processed by recombination enzymes. In several replication mutants, replication fork reversal is catalysed by the RuvAB complex, originally characterized for its role in the last steps of homologous recombination, branch migration and resolution of Holliday junctions. We present here the isolation and characterization of *ruvA* and *ruvB* single mutants that are impaired for RFR at forks arrested by the inactivation of polymerase III, while they remain capable of homologous recombination. The positions of the mutations in the proteins and the genetic properties of the mutants suggest that the mutations affect DNA binding, RuvA–RuvB interaction and/or RuvB-helicase activity. These results show that a partial RuvA or RuvB defect affects primarily RFR, implying that RFR is a more demanding reaction than Holliday junction resolution.

## Introduction

Chromosome replication is not a continuous process but can be impaired by obstacles or by the inactivation of a replication protein. Replication arrest can have dramatic consequences and replication defects are now recognized as a major source of genomic instability in all organisms (Michel, 2000; Branzei and Foiani, 2007; Lambert *et al*., 2007; Tourriere and Pasero, 2007). Consequently, restart of inactivated replication forks while limiting DNA damage and DNA recombination is a crucial process. It is now clear that a large diversity of enzymes will cooperate to rescue inactivated replication forks, depending on the cause of replication arrest. In *Escherichia coli*, in spite of the existence of a well-characterized multiprotein replication restart system, most often inactivated replication forks do not simply restart. A large panel of different reactions can take place prior to restart, which are determined by the cause of replication inactivation (Michel *et al*., 2004; 2007). A second important point is that recombination proteins are involved in all the reactions that were observed to take place prior to replication restart. Recombination proteins act on their normal substrates, double-strand DNA ends, single-strand DNA gaps or Holliday junctions (HJs), which are made at blocked forks, and they also act directly on replication forks, catalysing specific novel reactions (Baharoglu *et al*., 2006).

**Fig. 1:**
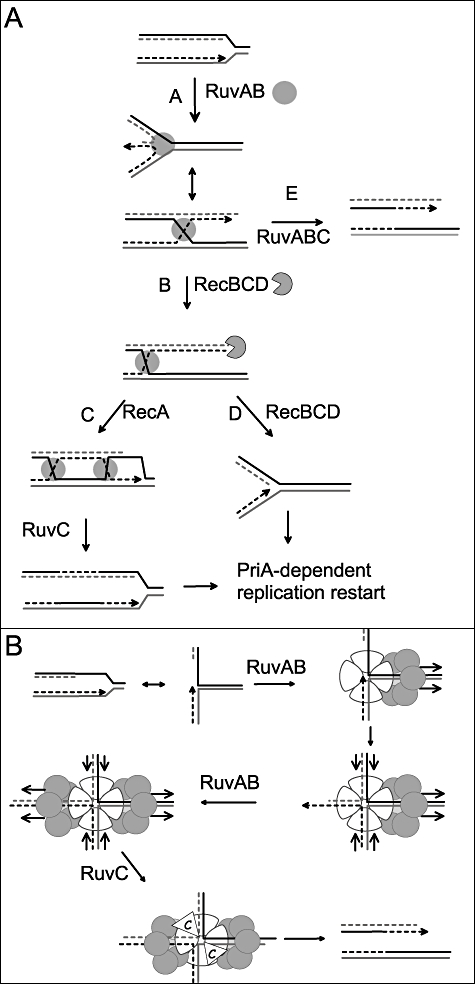
A. Model for replication fork reversal in a *dnaEts* mutant (adapted from Baharoglu *et al*., 2008). In the first step (A), the replication fork is arrested by inactivation of DnaE (the catalytic subunit of Pol III). RuvAB catalyses the annealing of leading and lagging strand ends, i.e. fork reversal. The reversed fork forms a four-arm structure (Holliday junction, HJ; two alternative representations of this structure are shown, open X and parallel stacked X). RecBC is essential for resetting of the fork, either by RecA-dependent homologous recombination (B–C) or by DNA degradation (B–D). In the absence of RecBCD (E), resolution of the HJ causes chromosome linearization. Continuous lines: parental chromosome strands. Dashed lines: newly synthesized strands. Circle: RuvAB. Incised circle: RecBCD. B.Model of RuvAB action at blocked forks. In the first step, RuvA binds to the fork and drives the assembly of a RuvB hexamer on the template strands (a tetramer of RuvA is drawn, although an octamer may be required to convert the fork into an HJ). The translocase action of this RuvB hexamer pulls the leading and lagging strands into the RuvA complex (direction of migration of DNA is indicated by arrows) and results in the formation of an HJ. This HJ is bound by a second RuvB hexamer forming a *bona fide* branch migration complex. HJ resolution by RuvC results in a cleaved replication fork.

In several replication mutants, a specific reaction takes place prior to replication restart, called replication fork reversal (RFR; Fig. 1A; Seigneur *et al*., 1998; Michel *et al*., 2007). Forks are reversed by the annealing of the leading and lagging strand ends, which results in the formation of an HJ adjacent to a DNA double-strand end. This DNA double-strand end is processed by RecBCD, the enzyme that initiates recombinational repair of DNA double-strand breaks in *E. coli* (Kowalczykowski, 2000). RecBCD is an exonuclease-recombinase and, at reversed forks, it either degrades the double-strand end or promotes its re-integration into the homologous chromosome by RecA-dependent recombination (Fig. 1A, pathway B). HJs are resolved in *E. coli* by the RuvABC complex (Yamada *et al*., 2004). RuvA and RuvB form a complex composed of one or two tetramers of RuvA associated with two hexamers of RuvB; this complex has been extensively characterized for its property of branch migration of HJs formed during homologous recombination (West, 1997). Associated with the endonuclease RuvC, it promotes HJ resolution. At reversed forks and in the absence of RecBC, resolution by RuvABC of the HJ formed by RFR leads to fork breakage (Fig. 1A, pathway E). Cells lacking RecBC are therefore used to assay RFR by measuring RuvABC-dependent fork breakage after replication inactivation.

The first step of RFR is the conversion of a three-arm fork structure into an HJ. This step is catalysed by different means in different replication mutants. It requires RecA in one mutant, the *dnaBts* mutant affected for the replicative helicase DnaB (Seigneur *et al*., 2000), whereas it is catalysed by RuvAB in *dnaEts* and *holD* mutants, affected for two different subunits of the main *E. coli* polymerase Pol III (Baharoglu *et al*., 2006) [DnaE is the Pol III catalytic subunit and HolD is a subunit of the clamp loader complex (O'Donnell, 2006)]. The occurrence of RuvAB-catalysed RFR implies that RuvAB recognizes blocked replication forks and is able to convert them into HJs (Fig. 1B). In order to characterize the action of RuvAB on its two different targets, HJs and replication forks, we screened for dissociation-of-function *ruvA* mutants that are specifically affected for RFR. We recently reported the isolation and characterization of two *ruvA* mutants that are fully proficient for homologous recombination but are unable to reverse *dnaEts*-blocked forks (Baharoglu *et al*., 2008). These mutants were obtained by PCR mutagenesis and carry multiple mutations. In the present work, we used an *in vivo* mutagenesis approach and isolated four single *ruvA* mutants and four single *ruvB* mutants which present a dissociation-of-function phenotype. Genetic characterization of these mutants shows that RFR-deficient *ruvA* or *ruvB* alleles encode partially impaired RuvAB complexes, supporting the idea that RFR is more demanding than recombination intermediates resolution.

## Results

### Isolation of Rec^*+*^/RFR-deficient mutants

**Fig. 2:**
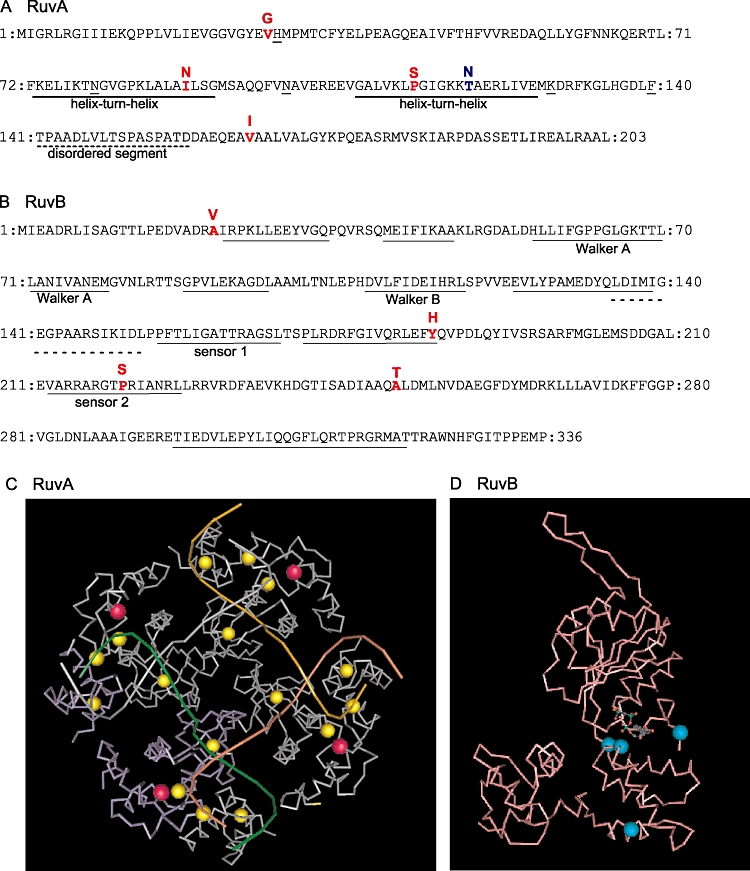
Positions of *ruvA* and *ruvB* mutations in the primary sequence. A.RuvA: Full and dashed lines indicate the positions of the two helix–turn–helix motifs in domain II and of the disordered segment that separates domains II and III respectively (Nishino *et al*., 1998). Domains I (1–64), II (65–140) and III (156–203) are not indicated. Mutations that impair RFR are in red with the replacing amino acid indicated above the sequence. The mutation T120N that suppresses the *ruvB* P220S mutant defect is in blue. Separation-of-function mutations previously isolated (N79D N100D and H29R K129E F140S) are underlined. B.RuvB: Full lines indicate the AAA motifs, and the dashed lines indicate the β-hairpin loop known to interact with RuvA (Iwasaki *et al*., 2000). Mutations are in red with the replacing amino acid indicated above the sequence. C.Three-dimensional ribbon structure of a RuvA tetramer viewed at the DNA binding face. Three monomers are in grey and one is in purple. Mutations that affect RFR are shown as yellow spheres and the T120N-suppressing mutation is shown as a pink sphere. D.Three-dimensional ribbon structure of a RuvB monomer (pink) bound to ADP (blue). Mutations that impair RFR are shown as blue spheres.

**Fig. 3:**
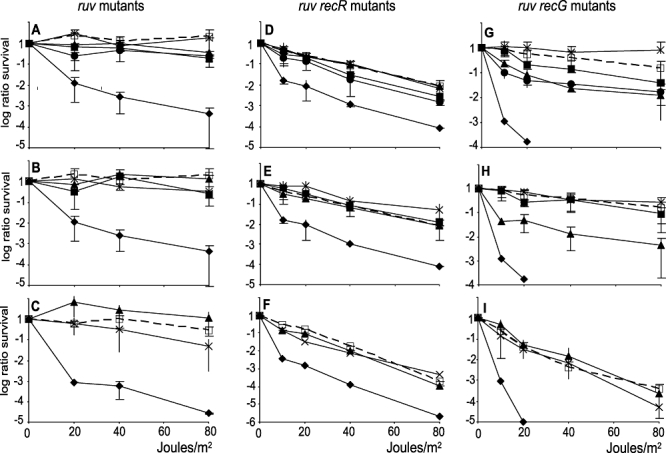
*ruvA* and *ruvB* mutants suppress the UV sensitivity of *ruvAB* or *ruvA* mutants. A–C. Suppression in *ruv* mutants. Appropriate dilutions of exponentially growing JJC 2907 (*ruvA*::Tn*10*, A and B) or JJC2971 (*ruvA100*, C) containing different plasmids were plated on LB spectinomycin, UV-irradiated and incubated overnight. Ratios of cfu on irradiated versus non-irradiated plates were calculated. Average of at least three values and standard deviations are shown. D–F. Suppression in *ruv recR* mutants. Same experiments with JJC4447 (*ruvA*::Tn*10 recR*; D and E) or JJC3375 (*ruvA100 recR*; F*)*. G–I. Suppression in *ruv recG* mutants. Same experiments in *ruvA*::Tn*10 recG* (G and H) or *ruvA100 recG*(I) cells. A, D and G. Closed diamonds: pGB2; open squares, dashed line: pGB-ruvAB (RuvA^+^ RuvB^+^); closed circles: pGB-RuvA^+^-RuvB-A250T; closed triangles: pGB-RuvA^+^-RuvB-P220S; closed squares: pGB-RuvA^+^-RuvB-Y184H; stars: pGB-RuvA^+^-RuvB-A22V. B, E and H. Closed diamonds: pGB2; open squares, dashed line: pGB-ruvAB; closed triangles: pGB-RuvA-I89N-RuvB^+^ closed squares pGB-RuvA-V28G-RuvB^+^ stars: pGB-RuvA-T120N-RuvB-P220S. C, F and I. Closed diamonds: pGB2; open squares, dashed line: pGB-ruvA (pGB-RuvA^+^); closed triangles: pGB-RuvA-V164I; stars: pGB-RuvA-P114S.

In order to mutate the *ruvA* and *ruvB* genes, the pGB-ruvAB plasmid was introduced into a *mutD5* mutator strain. *mutD*, also called *dnaQ*, encodes the proofreading subunit of Pol III. In its absence, lack of proofreading during DNA synthesis increases the rate of replication errors 10^4^-fold (Schaaper, 1993). Plasmids encoding a Rec^+^/RFR-deficient RuvAB complex were selected in a *dnaEts* context. The *dnaE486ts* mutant is killed at 42°C by the inactivation of the Pol III catalytic subunit DnaE. It grows at the semi-permissive temperature 37°C, but the impaired Pol III then leads to the formation of SOS-inducing gaps and arrested-restarting replication forks (Grompone *et al*., 2002; Lestini and Michel, 2007). Consequently, *dnaEts* cells are killed at 37°C by *ruvAB* inactivation because of unresolved HJs made by RecFOR-dependent gap repair, and they are also killed when *recB* is inactivated because of the occurrence of RFR (Baharoglu *et al*., 2006). The *dnaEts ruvAB recBCts* mutant, in which both *ruvAB* and *recBC* are inactive at 37°C, does not grow at 37°C because of *ruvAB* inactivation, but remains non-viable when *ruvA* and *ruvB* wild-type genes are introduced because RuvAB then catalyses RFR and RecBC is inactive. The only way to make a *dnaEts recBCts ruvAB* mutant viable at 37°C is to introduce *ruvAB* alleles that resolve HJs but do not catalyse RFR. To select such alleles, pGB-ruvAB plasmids extracted from six different *mutD5* clones were introduced in *dnaEts recBCts ruvAB* competent cells at 37°C. *dnaEts recBCts ruvAB* [pGB-RuvAB] transformants were obtained, and, in order to ascertain that the plasmids present in these clones carry *ruvAB* genes that were able to resolve HJs, we tested their ability to suppress the UV sensitivity of a *ruvAB* null mutant (this UV sensitivity results from the HJ resolution defect; Donaldson *et al*., 2006). Twenty-six plasmids that improved *dnaEts recBCts ruvAB* viability at 37°C were introduced into a *ruvAB* mutant; seven of them carried a mutation in *ruvA* and/or *ruvB* but still fully suppressed the sensitivity of a *ruvAB* mutant to UV irradiation. One carried a *ruvA* mutation (V28G), five carried a *ruvB* mutation (A22V, Y184H, A250T, P220S and P111L) and one carried two mutations, one in *ruvA* and one in *ruvB* (*ruvA*-T120N *ruvB*-P220S). The positions of the mutations in the *ruvA* and *ruvB* genes are shown in Fig. 2, and the UV resistance that they confer is shown in Fig. 3A and B. The same protocol was applied to a pGB-ruvA plasmid, yielding two plasmids that allow *dnaEts recBCts ruvA100* growth at 37°C and confer UV resistance to a *ruvA100* mutant. Each of these two plasmids carried a *ruvA* mutation (V164I and P114S; Figs 2 and 3C).

In another experiment, because we were concerned that the first screen was too stringent, pGB-ruvAB plasmids extracted from *mutD5* clones were also introduced in a *dnaEts recBCts lexAind ruvA* mutant. By preventing SOS induction, the *lexAind* mutation improves the viability of the *dnaEts* mutant at semi-permissive temperature (Grompone *et al*., 2002). Actually, more transformants were obtained in the *lexAind* context than in the LexA^+^*dnaEts recBCts ruvA* mutant (ratio of transformants at 37°C versus 30°C of about 5 × 10^−3^ instead of 10^−4^). These transformants were pooled for plasmid extractions and a genetic test that allows a direct selection of Rec^+^ plasmids was used to identify such plasmids in these extracts. The *dnaNts ruvAB* mutant, impaired for the Pol III clamp, is killed at semi-permissive temperature by unresolved RecFOR-dependent recombination events. Plasmids extracted at 37°C from 12 pools of *dnaEts recB ruvA lexAind* [pGB-ruvAB] transformants were introduced into the *dnaNts ruvAB* mutant at 37°C. Only one pool of plasmids, and only one plasmid in this pool, yielded *dnaNts ruvAB* clones able to propagate at 37°C, suggesting that the *lexAind* context provided a lot of fully inactivated *ruvAB* mutants. The only plasmid that allowed growth of *dnaNts ruvAB* cells at 37°C also suppressed the UV sensitivity of a *ruvAB* mutant. The *ruvA* and *ruvB* genes on this Rec^+^ plasmid were sequenced, showing a mutation in *ruvA* (I89N, Figs 2 and 3B).

### Mutant alleles are deficient or affected for RFR

In order to ascertain that the *ruvA* and *ruvB* mutant alleles that do not kill a *dnaEts recBCts ruvAB* mutant at 37°C are indeed deficient for RFR, the purified pGB-ruv(AB)m plasmids were re-introduced in the *dnaEts recBCts ruvAB* mutant and fork breakage was measured by pulse field gel electrophoresis [PFGE, pGB-ruv(AB)m stands for the seven pGB-ruvAB plasmids with a mutation in *ruvA*, *ruvB* or both]. Briefly, the chromosomes were labelled by growing cells in ^3^H-Thy-containing medium; cells were lysed in agarose plugs and run on pulse field gel (PFG). As only the linear DNA can enter PFG, the proportion of linear DNA *in vivo* was calculated as the ratio of DNA that enters gels versus total DNA in each migration lane. A high proportion of linear DNA is observed with *dnaEts recBCts* cells, owing to RuvAB-catalysed RFR and RuvABC resolution of the resulting HJ (60%, Grompone *et al*., 2002). The proportion of linear DNA is strongly decreased by *ruvAB* inactivation, which prevents fork reversal in the *dnaEts* mutant (10%, Grompone *et al*., 2002; Table 1). Introduction of wild-type *ruvAB* genes on pGB-ruvAB plasmid restores a high level of fork breakage, and introduction of the different pGB-ruv(AB)m alleles leads to variable levels of fork breakage (Table 1). The *ruvB*-P111L mutant was clearly capable of fork reversal and was not studied further (70% fork breakage, data not shown). Two *ruvB* mutations (*ruvB*-A22V and *ruvB*-A250T) and one *ruvA* mutation (*ruvA*-V28G) affected RFR partially (40–45% linear DNA). RFR was strongly decreased by the *ruvB*-Y184H mutation (23% linear DNA) and completely abolished by the *ruvA*-I89N and the *ruvB*-P220S mutations (about 10% linear DNA). Interestingly, the presence of the *ruvA*-T120N mutation restored fork breakage when combined with *ruvB*-P220S. This double mutant was obtained independently from the *ruvB*-P220S single mutant, raising the possibility that this *ruvB* mutation aroused twice independently and was, in one case, later compensated by the *ruvA*-T120N mutation.

**Table 1:** The *ruvA* and *ruvB* isolated alleles decrease fork breakage.

Strain	Relevant genotype	Plasmid	% linear DNA (*n*)
JJC2880	*dnaEts recBCts ruvAB*	None	7 ± 2.9 (8)
JJC2880	*dnaEts recBCts ruvAB*	pGB-RuvAB	71 ± 5.8 (8)
JJC2880	*dnaEts recBCts ruvAB*	pGB-RuvA^+^-*ruvB-*P220S	8.3 ± 2.4 (3)
JJC2880	*dnaEts recBCts ruvAB*	pGB-RuvA^+^-*ruvB*-Y184H	23 ± 2.3 (3)
JJC2880	*dnaEts recBCts ruvAB*	pGB-RuvA^+^-*ruvB*-A22V	46 ± 4.7 (3)
JJC2880	*dnaEts recBCts ruvAB*	pGB-RuvA^+^-*ruvB*-A250T	41 ± 6.3 (3)
JJC2880	*dnaEts recBCts ruvAB*	pGB-*ruvA*-I89N-RuvB^+^	10.5 ± 2.2 (3)
JJC2880	*dnaEts recBCts ruvAB*	pGB-*ruvA*-V28G-RuvB^+^	42 ± 4.3 (3)
JJC2880	*dnaEts recBCts ruvAB*	pGB-*ruvA*-T120N*-ruvB*-P220S	71.5 ± 2.8 (3)
JJC3723	*dnaEts recBCts ruvA100*	None	7 ± 1.5 (4)
JJC3723	*dnaEts recBCts ruvA100*	pGB-RuvA	55.3 ± 3.2 (3)
JJC3723	*dnaEts recBCts ruvA100*	pGB-*ruvA*-V164I	27 ± 3.4 (3)
JJC3723	*dnaEts recBCts ruvA100*	pGB-*ruvA*-P114S	31 ± 6.1 (3)
JJC3723	*dnaEts recBCts ruvA100*	pGB-*ruvA*-V164I-RuvB^+^	70.6 ± 1.5 (3)
JJC3723	*dnaEts recBCts ruvA100*	pGB-*ruvA*-P114S-RuvB^+^	59.4 ± 3.3 (3)

*n* is the number of independent experiments.

Similarly, fork breakage was measured in *dnaEts recBCts ruvA100* cells carrying pGB-ruvAm mutant plasmids. For both pGB-ruvAm mutants, RFR remained strongly decreased, although not fully abolished (27–31% fork breakage, Table 1). *ruvA* and *ruvB* genes form an operon, with *ruvB* downstream of *ruvA*. *ruvB* expression is prevented by the *ruvA60*::Tn*10* mutation used here (called here *ruvAB*, it is not complemented by the expression of *ruvA* only, data not shown), whereas the Cm^R^ insertion in the *ruvA100* allele affects but does not prevent the expression of the downstream *ruvB* gene (called here *ruvA*, it is complemented by the expression of *ruvA* only, Baharoglu *et al*., 2008). The two *ruvA* dissociation-of-function mutants previously characterized (N79D N100D and H29R K129E F140S) affect RuvA–RuvB interactions *in vitro*, and were only deficient when *ruvB* was expressed downstream of the *ruvA100* allele, as they became proficient for RFR when *ruvB* was coexpressed with the *ruvA* mutant allele, from the plasmid or from the chromosome (Baharoglu *et al*., 2008). In order to test whether coexpression of *ruvB* suppresses the RFR defect of *ruvA*-V164I and *ruvA*-P114S mutants, these *ruvA* mutations were introduced by site-directed mutagenesis on the pGB-ruvAB plasmid. Fork breakage in *dnaEts recB ruvAB* cells was shown to be restored to a high level in the presence of the pGB-ruvAm-ruvB^+^ plasmids (Table 1). This result indicates that the two RFR-deficient *ruvA* mutants isolated on a plasmid that does not carry *ruvB* become capable of RFR if the level of RuvB protein is increased.

Altogether, we have identified four *ruvA* and four *ruvB* mutations that impair or abolish RFR. For the two *ruvA* mutants isolated on a plasmid that does not carry *ruvB*, RFR is only decreased if RuvB is expressed from the chromosome locus downstream of the *ruvA100* mutation. Interestingly, a *ruvB* mutation (P220S) was isolated twice, once alone where it prevents RFR, and once combined with a *ruvA* mutation that restores RFR.

### The RFR-deficient mutants complement the UV sensitivity of *ruvA(B)* mutant

All RFR mutants restore full resistance to UV irradiation in *ruvAB* or *ruvA* mutants (Fig. 3A–C). As the role of RuvAB post UV irradiation is the resolution of recombination intermediates, the resistance to UV irradiation is a strong indication that these *ruvA* and *ruvB* alleles are capable of HJ resolution *in vivo*. We further tested the recombination proficiency of these alleles in combination with other recombination mutations and by other assays.

The UV sensitivity due to *ruvAB* inactivation is synergistic with the inactivation of *recFOR*, which prevents gap repair (Baharoglu *et al*., 2008; Fig. 3, pGB2-containing strains). All pGB-ruv(AB)m plasmids suppressed the *ruvAB* defect in a *ruvAB recR* double mutant (Fig. 3D and E). Similarly, the two pGB-ruvAm plasmids suppressed the *ruvA100* repair defect in a *ruvA100 recR* mutant, to the same extent as pGB-ruvA (Fig. 3F). In conclusion, these experiments show that all *ruvA* and *ruvB* mutations isolated do not affect the capacity of RuvAB to resolve HJs during recombinational repair of UV DNA lesions, in *recR* as well as in Rec^+^ contexts.

Residual recombination proficiency in *ruv* null mutants depends on the integrity of the *recG* gene, which encodes a helicase capable of HJ branch migration *in vitro* (Lloyd, 1991; Whitby and Lloyd, 1998). The *recG* mutation was introduced by P1 transduction in *ruvAB* cells containing pGB-ruv(AB)m and in *ruvA100* cells containing pGB-ruvAm. First, the presence of these plasmids suppressed the poor viability of *ruvAB recG* and *ruvA recG* double mutants (data not shown). Second, compared with the *ruv recG* double mutant that carries the vector (pGB2), all plasmids suppressed most or all the UV sensitivity of *ruvA recG*, or *ruvAB recG* mutants (Fig. 3G and H). Only a residual UV sensitivity was observed with some plasmids (*ruvB*-A250T, *ruvB*-P220S and *ruvA*-I89N).

Both pGB-ruvA-P114S and pGB-ruvA-V164I suppressed the UV sensitivity of *ruvA100 recG* cells as efficiently as pGB-ruvA (Fig. 3I). It was observed that *ruvA100 recG* [pGB-ruvA] cells were significantly more UV-sensitive than *ruvAB recG* [pGB-ruvAB] (compare Fig. 3G–I); the *ruvA100 recG* [pGB-ruvA] defect results from the limiting amount of RuvB in this mutant as it was fully suppressed by pGB-ruvAB wild type, and by the plasmids expressing RuvB together with *ruvA*-P114S or *ruvA*-V164I (data not shown).

### Some, but not all RFR-deficient mutants complement the mitomycin C sensitivity of *ruvA(B)* mutant

**Fig. 4:**
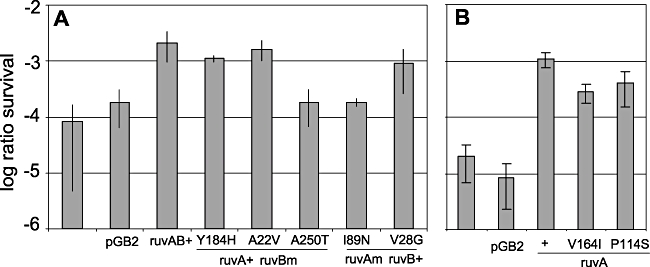
Most *ruvA* and *ruvB* mutants alleles suppress the mitomycin C sensitivity of *ruvAB* or *ruvA* null mutants. Exponentially growing JJC 2907 (*ruvA*::Tn*10*, A) or JJC 2971 (*ruvA100*, B) containing different plasmids was treated with 2 μg ml^−1^ mitomycin C for 90 min, plated on LB spectinomycin and incubated overnight. Ratios of colony-forming units (cfu) in treated versus untreated cultures are shown.

Mitomycin C is a DNA-damaging agent that introduces a variety of lesions, including interstrand cross-links and base monoadducts (Keller *et al*., 2001). These DNA lesions are in part repaired by homologous recombination, rendering *ruvAB* mutants sensitive to mitomycin C (Shurvinton and Lloyd, 1982; Baharoglu *et al*., 2008). In our experimental conditions, the *ruvAB* mutant was 10- to 20-fold more sensitive to mitomycin C than the wild-type strain (not shown) or than the *ruvAB* mutant carrying pGB-ruvAB^+^ (Fig. 4A). Two *ruvB* (A22V, Y184H) and one *ruvA* (A28G) mutant alleles suppressed the mitomycin C sensitivity of the *ruvAB* null mutant as efficiently as the *ruvAB* wild-type gene, RuvB-A22V, RuvB-Y184H and RuvA-A28G. One *ruvB* and one *ruvA* mutant alleles did not suppress the mitomycin C sensitivity of the *ruvAB* mutant: *ruvB*-A250T and *ruvA*-I89N (Fig. 4A). For unknown reasons, results with *ruvB*-P220S and with the *ruvA*-T120N *ruvB*-P220S double mutant were highly variable and could not be interpreted (six independent experiments, data not shown). These results confirm that certain mutants are fully recombination proficient and indicate that two of them, although they fully suppress the UV sensitivity of *ruvAB* null mutants (Fig. 3), have retained some recombination deficiency in mitomycin C-treated cells.

The *ruvA100* mutant is 100-fold more sensitive to mitomycin C than the wild-type strain (Fig. 4B). For unknown reasons, *ruvA100* [pGB2] cells were about 10-fold more sensitive to UV irradiation and to mitomycin C treatment than *ruvAB* [pGB2] cells, suggesting that RuvB expressed downstream of the *ruvA100* allele might affect HJ resolution in the absence of RuvA. We tested the capacity of *ruvA* single mutants to suppress the repair defect of the *ruvA100* mutant and observed that the two *ruvA*-V164I and *ruvA*-P114S mutations conferred a nearly wild-type level of survival to mitomycin C (Fig. 4B); therefore, these alleles are able to promote HJ resolution during the recombinational repair of mitomycin C DNA lesions.

### The RFR-deficient mutants complement the conjugational recombination defect of a *ruvA(B) recG* double mutant

**Fig. 5:**
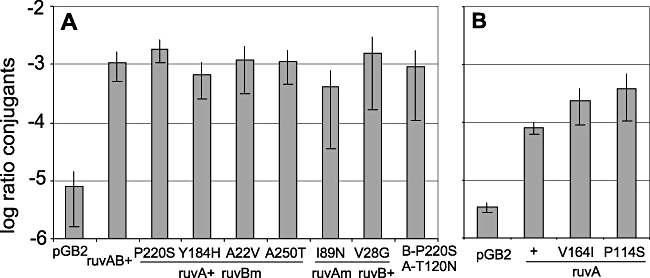
*ruvA* and *ruvB* mutants suppress the conjugational recombination defect of *ruvAB* or *ruvA* mutants in a *recG* context. Exponentially growing cells were mixed with a His^+^ Hfr donor for 25 min, plated on Kan (A) or Cm (B) minimal medium plates devoid of histidine and incubated for 48 h. Ratios of His^+^ versus total recipient cfu are shown. Recipient: (A) *ruvA::*Tn*10 recG*::*kan*^R^, (B) *ruvA100 recG*::*kan*^R^.

Homologous recombination on non-damaged DNA was tested by measuring Hfr conjugation efficiency. During Hfr conjugation, the chromosome of the donor cell is introduced in the recipient cell and ex-conjugants are obtained when the incoming chromosome recombines with the homologous sequence in the recipient chromosome. Hfr conjugation efficiency is only weakly affected by the inactivation of *ruvAB*, but nearly completely abolished in a *ruvAB recG* double mutant (Lloyd, 1991; Baharoglu *et al*., 2008). We compared Hfr conjugation efficiencies using as recipient cells *ruvAB recG* double mutants that contain either the pGB2 vector, or pGB-ruvAB, or one of the pGB-ruv(AB)m plasmid. As shown in Fig. 5A, the pGB-ruv(AB)m plasmids restored Hfr conjugational recombination. Similarly, Hfr conjugation was also restored in *ruvA100 recG* cells by the two pGB-ruvAm plasmids (Fig. 5B). Altogether, these experiments show that the *ruvA* and *ruvB* mutant alleles that decrease or abolish RFR in the *dnaEts* mutant are not significantly affected for homologous recombination of intact DNA.

### The *ruvAB* mutants are capable of preventing RusA action

**Fig. 6:**
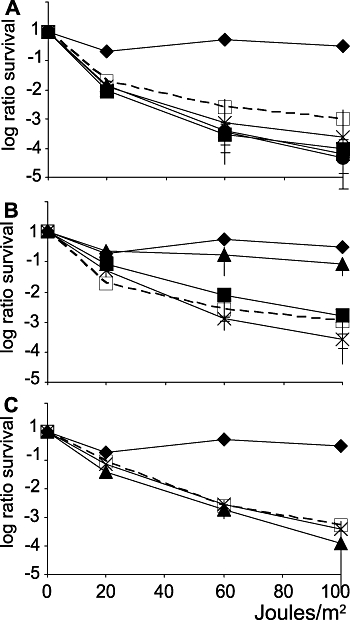
All plasmids but pGB-RuvA-I89N-RuvB^+^ prevent resolution of HJ by RusA. Same experiments as in Fig. 3 with JJC2761 (*ruvABC::cm rus-1*). Symbols are as in Fig. 3.

The *rusA* gene belongs to a cryptic prophage and encodes an HJ resolvase. This promoter-less gene is not expressed in *E. coli*, unless it is activated by the insertion of an upstream functional promoter, as in the *rus-1* allele (Mandal *et al*., 1993). The *rus-1* mutation suppresses the recombinational defects of *ruvAB* and *ruvABC* mutants, but RusA cannot act in a *ruvB* or *ruvC* mutant, owing to RuvA or RuvAB masking the HJ. Therefore, the capacity of RuvA alleles to recognize and bind HJs *in vivo* can be deduced from their capacity to prevent RusA action. pGB-ruv(AB)m and pGB-ruvAm plasmids were introduced into a *ruvABC rus-1* mutant and UV sensitivity was measured. As expected, in the presence of the vector pGB2, *ruvAB rus-1* cells were UV-resistant because of RusA-catalysed HJ resolution. None of the *ruvB* mutations prevented RuvA from sequestering the HJ (Fig. 6A). In contrast, with the exception of the *ruvA*-I89N mutant, all mutant *ruvA* alleles had retained the property of preventing RusA-mediated resolution, as they all rendered *ruvABC rus-1* cells sensitive to UV irradiation, similar to the wild-type RuvA protein (Fig. 6B and C). These results indicate that the *ruvA*-I89N mutation affects RuvA binding to HJs *in vivo*, while all other mutants bind HJs efficiently enough to prevent RusA action.

## Discussion

In this work we isolated several *ruvA* and *ruvB* mutant alleles that are much more affected for RFR than for homologous recombination. This work shows that mutations in various domains of RuvA or in RuvB separate the fork reversal function of RuvAB from its function as an HJ branch migration and resolution enzyme, suggesting that there are several ways of separating these two functions. The phenotypes of the different mutants are summarized in Table 2.

**Table 2:** Summary of the phenotypes of *ruvA* and *ruvB* RFR-deficient alleles.

	UV^R^ in wt and *recR*	UV^R^ in *recG*	UV^R^ in *rus-1*	MMC^R^	Hfr in *recG*	Fork breakage
pGB-ruvAB^+^	R	R	S	R	1	70%
ruvB-P220S	R	Weakly S	S	Variable	1	10%
ruvB-Y184H	R	R	S	R	1	23%
ruvB-A22V	R	R	S	R	1	46%
ruvB-A250T	R	Weakly S	S	S	1	41%
ruvA-I89N	R	Weakly S	R	S	1	10%
ruvA-V28G	R	R	S	R	1	42%
ruvB-P220S ruvA-T120N	R	R	S	Variable	1	70%
pGB-ruvA^+^	R	R	S	R	1	55%
ruvA-V164I	R	R	S	R	1	27%
ruvA-P114S	R	R	S	R	1	31%

MMC: mitomycin C; Hfr: Hfr conjugation; Fork breakage: percentage of linear DNA in a *dnaEts recB ruvA(B)* background (cf. Table 1). R: resistant; S: sensitive.

RuvA is composed of three domains. Domains I [amino acids (AA) 1–64] and II (AA 65–140) are involved in DNA binding while domain III (AA 155–203) is involved in RuvB binding. Domains II and III are separated by an unstructured flexible linker of 15 amino acids (Nishino *et al*., 1998; Rafferty *et al*., 1998; Ariyoshi *et al*., 2000; Yamada *et al*., 2002). The two *ruvA* separation-of-function mutants isolated previously were multiple mutants (Baharoglu *et al*., 2008; Fig. 2). Both mutant proteins were shown to bind HJ *in vitro* fairly well, but to be defective for octamerization (binding of two RuvA tetramers that sandwich a junction) and for binding to DNA fork structures. In addition, the triple mutant, H29R K129E F140S, was significantly affected for RuvB-helicase stimulation. These *ruvA* mutations were isolated on a plasmid that carries only *ruvA*, with *ruvB* expressed downstream of *ruvA100* in the chromosome. The *ruvA*-V164I and *ruvA*-P114S mutations were isolated here on the same plasmid and, as observed for the two previously characterized mutants, their RFR defect is suppressed when RuvB expression is increased (Table 1, plasmids pGB-*ruvA*-V164I-RuvB^+^ and pGB-*ruvA*-P114S-RuvB^+^). This observation suggests that these mutants suffer from a defect in RuvA–RuvB interactions, which has detectable consequences only when RuvB is in a limiting amount. The *ruvA*-V164I mutation lies within RuvA domain III, which interacts with RuvB (Fig. 2), and two mutations (L167A and L170A) within the same alpha helix as V164 (alpha 7) were previously shown to abolish RuvB activity *in vivo* (these two mutants are deficient for homologous recombination) without affecting RuvA binding to HJs *in vitro* (Nishino *et al*., 1998). *ruvA*-P114S is just before the flexible linker that separates domains II and III (Fig. 2). In support of the idea that this mutation also affects RuvB activation, the T120N mutation, only six amino acids distant from P114S, suppresses the RFR defect of the *ruvB*-P220S mutant. The helix from amino acids 118–130 is involved in interactions between two RuvA tetramers (Roe *et al*., 1998; Privezentzev *et al*., 2005; Fujiwara *et al*., 2008). Our observations suggest that mutations in this helix–turn–helix region of RuvA domain II also affect RuvA–RuvB interactions. Actually, a *Thermus thermophilus ruvA* mutant impaired for octamerization forms complexes in which a RuvA tetramer is flanked by two RuvB hexamers on an HJ, but these complexes exhibit impaired branch migration (Fujiwara *et al*., 2008; Mayanagi *et al*., 2008). In conclusion, the phenotypes of the P114S and V164I mutants suggest that impairing RuvA–RuvB interactions is sufficient to significantly decrease RFR without any detectable effect on homologous recombination. This observation supports a model in which RuvA–RuvB interactions are crucial for the stability and/or the action of RuvAB at forks.

Two other *ruvA* mutants were isolated on plasmids that carry both *ruvA* and *ruvB*. In contrast with the two *ruvA* mutants suppressed by RuvB coexpression, they both map in the N-terminal half of the RuvA protein, in proximity to the DNA, at positions 28 and 89 (Fig. 2C). The V28G mutation is adjacent to one of the mutations in the previously isolated triple mutant (H29R). This mutant is partially impaired for RFR, as fork breakage is only down to 42%, and is fully recombination proficient in all assays. Although the RuvA-V28G remains fully capable of preventing RusA action, given the position of the V28 and H29 residues on the tri-dimensional structure of RuvA, close to the crossing strands at the junction (Fig. 2C), it is likely that these mutations affect DNA contacts. The I89N mutation is in a helix–turn–helix motif, between the N79D and N100D mutations previously shown to prevent octamerization and fork binding (Baharoglu *et al*., 2008). The position of the mutation in the protein and the *in vitro* properties of the N79D N100D double mutant suggest that the primary defects conferred by the I89N mutation might also be DNA binding and RuvA octamerization. Accordingly, even when coexpressed with RuvB, this mutant is unable to prevent RusA action *in vivo*, an observation that suggests either that one face of the HJ remains accessible to RusA within the RuvA-I89N–HJ complex, or that RuvA-I89N easily dissociates from HJs. The I89N mutation has dramatic consequences, as it causes a drastic drop of fork breakage (8%), and shows sign of a slight recombination defect: it is slightly affected in the *recG* context for UV repair and remains sensitive to mitomycin C. This recombinational repair defect might reflect impairment of branch migration through certain DNA lesions. It is noteworthy that this mutant was isolated in the *lexAind* context, in a screen that yielded a lot of recombination-deficient (presumably null) mutants. Nevertheless, the *ruvA*-I89N mutant is recombination-proficient on intact DNA (Hfr conjugation) and in UV-irradiated cells, in which it only weakly needs RecG. The minor defects in HJ resolution cannot be the cause of the very low level of fork breakage, and the properties of this mutant support the idea that strong DNA binding, and possibly octamer formation, is needed for RuvA action at forks, logically because several DNA strands are missing in a RuvA–fork complex compared with a RuvA–HJ complex, and because octamerization, as in *T. thermophilus*, may stimulate RuvB activity (Fujiwara *et al*., 2008).

Four *ruvB* mutants were isolated in this work. The RuvB protein is composed of three domains (N, M and C), which form a crescent-like architecture (Putnam *et al*., 2001; Yamada *et al*., 2002; 2004; Ohnishi *et al*., 2005). The N and M domains are involved in ATP binding and hydrolysis and adopt the canonical fold of AAA+ family proteins (Ogura and Wilkinson, 2001). The N domain also contains a protruding β-hairpin (L135–L152) which interacts with RuvA (Han *et al*., 2001). The *ruvB*-A250T mutation lies in domain C (T241 to terminus), adjacent to a conserved leucine. It is the only mutant which is only partially affected for fork breakage and shows a slight recombination deficiency by all assays, except UV sensitivity in Rec^+^ and RecR contexts. It is conceivable that for this mutant the decrease in fork breakage results from the impairment for HJ resolution. The other three *ruvB* mutants behave as dissociation-of-function mutants, as they are much more strongly affected for RFR than for homologous recombination. They are all in the N or M domains of RuvB, adjacent or within one of the AAA conserved motifs (Fig. 2). The Ala22 residue, changed here to a valine, is not conserved in other bacterial RuvB proteins but is adjacent to the first conserved AAA motif. Changing the nearby Arg24 to cysteine or histidine inactivates the protein (Iwasaki *et al*., 2000). The Tyr184 residue, mutated here to histidine, is invariant in all bacterial RuvB proteins, within the AAA motif 8, and part of the ATP binding site (Iwasaki *et al*., 2000; Putnam *et al*., 2001). The Y184A mutation inactivates RuvB (Iwasaki *et al*., 2000). The Pro220 residue, changed here to a serine, is nearly invariant and also part of the ATP binding site, within the conserved sensor 2 motif of AAA proteins (Iwasaki *et al*., 2000; Putnam *et al*., 2001). Changing either of the flanking T219 or R221 residues to an alanine largely or fully inactivate RuvB (Iwasaki *et al*., 2000). It is surprising that the T184H and P220S mutations only very slightly affect homologous recombination. Given their positions in the protein, close to the ATP binding site (Fig. 2), these three *ruvB* mutations are likely to impair ATP binding or hydrolysis, suggesting that full RuvB ATPase activity is more essential for RFR than for homologous recombination, an observation in agreement with a model in which only one RuvB hexamer is present at forks. Interestingly, the P220S mutation is fully suppressed by the RuvA T120N mutation. It suggests that the alpha 6 helix of RuvA (AA 118–129) could be involved in long-range communications between RuvA, RuvB and DNA. This helix is part of the helix–turn–helix motif and is involved in contacts between two RuvA tetramers, while the suppressed *ruvB* P220S mutation is in one of the ATPase sensor motifs. It is tempting to speculate that coupling of ATP hydrolysis and branch migration involves interactions through the tetramerization helix of RuvA (T120) when bound to DNA, and the ATP sensing of RuvB (P220) and DNA.

In conclusion, this work shows that there are multiple ways of decreasing the activity of the RuvAB complex so that it remains capable of producing recombinant molecules with a normal or close to normal efficiency, while being largely or fully inactivated for its capacity to reverse blocked forks.

## Experimental procedures

### Strains and plasmids

Strains are described in Table 3. Mutations were introduced by P1 transduction. For the construction of plasmid-containing strains, the plasmids were introduced at the last step by transformation of CaCl_2_ competent cells. A different protocol was used for the *ruv recG* double mutants. As these mutants are very sick and therefore difficult to make competent, plasmid-containing *ruv recG* double mutants were constructed from plasmid-containing *ruv* single mutants, by P1 transduction of the *recG253*::*kan* mutation. Fresh *recG* transductants were constructed for each independent experiment. Antibiotics were used at the following concentration: spectinomycin (Spc) 60 μg ml^−1^, chloramphenicol (Cm) 20 μg ml^−1^, kanamycin (Kan) 50 μg ml^−1^, tetracycline (Tet) 15 μg ml^−1^. The presence of the *mutD5* mutation was tested by measuring the ratio of Rif^R^ spontaneous mutants in overnight cultures (about 10E^−4^ in the *mutD5* mutant versus 10E^−8^ in the wild-type strain, using rifampicin 50 μg ml^−1^). Sequencing of *ruvA* and *ruvB* genes in plasmids was performed using ‘Genetic Analyser’ 3100 (Applied Biosystem) automatic sequencer. The *ruvA ruvB* operon was amplified with oligonucleotides pGB1: CGAAGTAATCGCAACATCCGC and pGBa: CAATATGTGTCCCGACCCTAG. These oligonucleotides were also used for sequencing as well as the following ones: ruvA: GCTTCCTAGGGGCCCTTAA and CTTCCGGCAAAGTGGTACC; ruvB: GTCCCGCTCGCTAAAACGAG, GCGGCCTGGAAGTGGTTAGT, CGTCGGTGGTGCTATGTGCG and ACGAGCAACTTCCAGCGCGC.

**Table 3:** Strains.

Strain	Relevant genotype	Construction or reference
JJC40	Wild type	As AB1157 but *hsdR* Thr^+^ Pro^+^
JJC145	Hfr PK19-PO66 Δ-(*gpt-lac*) *supE44 srlC::*Tn*10 thi1*	CGSC6813 Genetic Stock Center
JJC720	*mutD5*	Schaaper (1993)
JJC944	Δ*recG263*::kan	N3793 = CF3324 in Mandal *et al*. (1993)
JJC1193	*recR252*::Tn*10*kan	Baharoglu *et al*. (2008)
JJC2211	*sfiA11 recB270ts recC271ts dnaE486ts zae3095*::Tn*10*kan	Lestini and Michel (2007)
JJC2434	*sfiA11 dnaN159ts zid501*::*Tn*10	Lestini and Michel (2007)
JJC2761	*sfiA11*Δ*ruvABC::Cm rus-1*	Baharoglu *et al*. (2006)
JJC2818	*sfiA11 dnaE486ts zae3095*::Tn*10*kan	Flores *et al*. (2005)
JJC2880	*sfiA11 recB270ts recC271ts dnaE486ts zae3095*::Tn*10*kan *ruvA60*::Tn*10*	JJC2211 * P1 JJC 2907
JJC2907	*ruvA60*::Tn*10*	JJC40 * P1 N2057 Shurvinton *et al*. (1984)
JJC2971	Δ*ruvA100::*Cm	Baharoglu *et al*. (2008)
JJC3192	*sfiA11 dnaN59ts zid*::Tn*10*Δ*ruvA100::*Cm	JJC2434 * P1 JJC2971
JJC3207	Δ*ruvA100::*Cm Δ*recG263*::kan	Baharoglu *et al*. (2008)
JJC3297	*sfiA11 dnaE486ts zae3095*::Tn*10*kan *lexAind mal*::Tn*10*Δ*recB*::*Ap*Δ*ruvA100::*Cm	JJC2818 * P1 *lexAind mal*::Tn10* P1 JJC2971 * P1 Δ*recB*::*Ap*
JJC3375	Δ*ruvA100::*Cm *recR*::kan	Baharoglu *et al*. (2008)
JJC3723	*sfiA11 recB270ts recC271ts dnaE486ts zae3095*::Tn*10*kan Δ*ruvA100::*Cm	Baharoglu *et al*. (2008)
JJC4447	*ruvA60*::Tn*10 recR252*::Tn*10*kan	JJC2907 * P1 JJC1193

### UV and mitomycin C resistance tests

UV irradiation was performed as described (Baharoglu *et al*., 2006). For mitomycin C treatment, cells were grown at 37°C in LB to an OD_650_ = 0.5, mitomycin C was added to the culture at a final concentration of 2 μg ml^−1^ and incubation continued at 37°C for 90 min. An untreated culture was used as control. Appropriate dilutions were plated on LB plates and incubated overnight at 37°C (Baharoglu *et al*., 2008). Ratios of colony-forming units (cfu) of mitomycin C-treated over cfu of untreated cells were calculated.

### Conjugation

Conjugations were performed as described using JJC145 as Hfr donor (Lestini and Michel, 2007). Donor and recipient cells were mixed for 25 min. Selective medium was M9 minimal medium supplemented with leucine, proline, threonine and arginine (2% final concentration each), and 10 μg ml^−1^ Cm for *ruvA100 recG*::Kan recipient cells, 10 μg ml^−1^ Kan for *ruvA60*::Tn*10 recG*::Kan recipient cells.

### Measurement of linear DNA by PFGE

Quantification of pulsed field gels was performed using *in vivo*^3^H-thymidine labelled chromosomes as previously described (Seigneur *et al*., 1998).
